# Evolution of Clinical Trials in Ovarian Cancer Management over the Past 20 Years: Never Settle Down, Always Go Beyond

**DOI:** 10.1155/2021/1682532

**Published:** 2021-10-07

**Authors:** Francesca De Felice, Laura Vertechy, Elena Giudice, Raffaella Ergasti, Serena Boccia, Anna Fagotti, Giovanni Scambia, Claudia Marchetti

**Affiliations:** ^1^Department of Radiotherapy, Policlinico Umberto I, Sapienza University of Rome, Rome, Italy; ^2^Department of Woman, Child and Public Health, Fondazione Policlinico Universitario A. Gemelli IRCCS, Rome, Italy; ^3^Institute of Obstetrics and Gynecology, Università Cattolica Del Sacro Cuore, Rome, Italy

## Abstract

**Purpose:**

A practice synthesis of available evidence-based medicine data in ovarian cancer (OC), aiming to provide directions for future research.

**Materials and Methods:**

We performed a systematic review. PubMed was searched for relevant OC trials between January 2000 and December 2019.

**Results:**

Out of 865 references screened, 199 trials were found eligible for inclusion. Most trials were multicenter (83.9%). There was a trend reduction in the number of patients enrolled/per study over the years. Studies testing targeted/biological therapies dominated the second decade (60 trials in 2010–2019 versus 2 trials in 2000–2009). The proportion of trials with positive survival and clinical outcomes significantly increased from 23.8% in early 2000s to 54.1% in the last 5 years. Trials with histology/molecular biomarker criteria were more likely to meet progression-free survival endpoint than those without these selection criteria (69.2% versus 32.6%).

**Conclusion:**

This systematic review suggests a trend of increased positive studies, mainly linked to precision medicine.

## 1. Introduction

Despite substantial progresses in its understanding and treatment, OC is still a significant cause of mortality worldwide, accounting more than 150,000 estimated deaths per year [1]. A steadily growing body of scientific publications on this topic has been recorded over the past 20 years, leading to changes in treatment toward a patient-tailored approach [2]. The evidence-based medicine (EBM) approach is a method used to establish the most relevant studies able to have an impact on clinical practice. EBM defines the concept of integration of best research evidence with clinical expertise and patient preferences [3]. It is based on the best available clinical evidence from systematic research using the medical literature, and results of randomized controlled trials are considered the top in the hierarchy of research designs evidence [3]. Here, we provide insight into randomized clinical trials that have been performed in the last 20 years.

## 2. Methods

### 2.1. Data Sources and Outcomes

We systematically reviewed the literature. Literature search was performed according to the Preferred Reporting Items for Systematic Reviews and Meta-Analyses (PRISMA) guidelines [4]. PubMed database was searched for relevant citations published from January 2000 to December 2019, using the following keywords: “ovarian” AND “cancer(s) or neoplasm(s)” AND “therapy or treatment or therapeutics” limiting to randomized controlled trials written in English language. A total of 865 references have been identified over the past 20 years. Two independent reviewers screened the titles of studies and abstracts for relevance to the topic in consideration and subsequently reviewed the full text articles for inclusion. Disagreements were resolved by a third reviewer. When more than one article reported results with overlapping data, the reviewers chose the most recent or comprehensive. Ongoing trials, study design protocols, and phase I studies were not included. The following parameters were recorded into a standardized database: document title, author(s) surname, journal and year of publication, sample size, primary outcome, and results of the study (in term of difference/no difference between treatment groups). Primary outcomes were considered positive if they demonstrated statistically significant improvement (*p* value ≤ 0.05).

### 2.2. Statistical Analysis

A descriptive analysis was performed to provide an overview of the landscape of phase II and phase III ovarian cancer clinical trials. To facilitate clarity and understanding, four timeframes, 2000–2004, 2005–2009, 2010–2014, and 2015–2019, were considered. Changes over the five-year timeframes were analyzed by the linear-by-linear association trend test. Percentages were compared by the *χ*^2^ test (with appropriate number of degrees of freedom, depending on contingency table size). The *α* level (type I error) was set at 0.05. All data were analyzed using SPSS software.

## 3. Results

**Figure 1:**
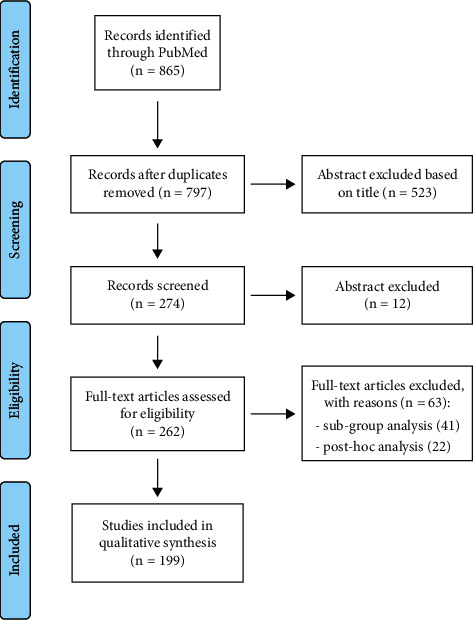
Flow diagram.

After an initial screening of titles and abstracts for relevance to the topic in consideration (*n* = 865), 274 studies were identified. Ongoing trials, study design protocols, and phase I studies were deemed not eligible (*n* = 12). Sixty-three additional studies were excluded because they were only subgroup analysis and/or post hoc analysis of trials just included in the review (we only considered the published studies concerning the primary and secondary outcome of the trial). A total number of 199 clinical trials were finally reviewed on the basis of their relevance into OC treatment (Figure 1). A total of 72934 patients were enrolled in 199 trials, and sample sizes ranged from 24 to 2074 patients (mean and standard deviation, 367 ± 375 patients). Details are listed in Table 1.

**Figure 2:**
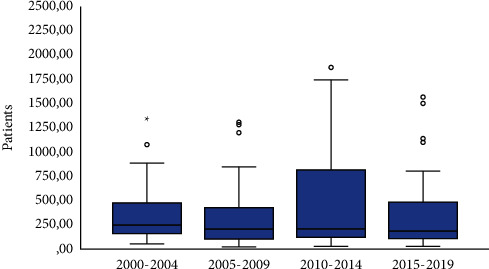
Box plot: number of patients enrolled/per study over time.

**Figure 3:**
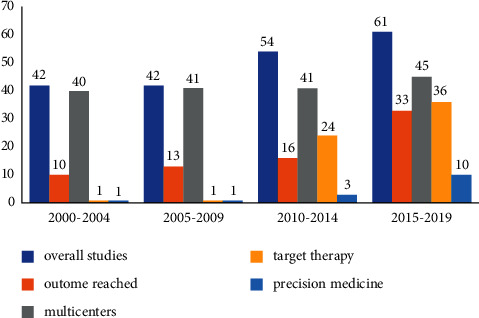
Trial characteristics over time.

Despite the difference was not statistically significant, there was a trend reduction in the number of patients enrolled/per study from the early 2000s (mean and standard deviation, 385 ± 369 patients) until 2015–2019 (mean and standard deviation, 323 ± 339 patients) (Figure 2). Studies frequency significantly increased from 42 studies in 2000–2004 to 61 studies in the last timeframe (2015–2019) (Figure 3).

In total, 12 trials (6.0%) representing 4742 patients (mean and standard deviation, 395 ± 188 patients) focused upon surgical interventions. A total of 45095 patients (mean and standard deviation, 480 ± 463 patients) were enrolled in 94 trials (47.2%) testing chemotherapy and maintenance therapy in the primary setting, and 23097 patients (mean and standard deviation, 248 ± 232 patients) were identified in 93 trials in the recurrent setting.

The absolute number of phase III trials progressively increased from 22 in 2000–2004 to 29 in 2015–2019. Examining the proportion of phase III trials/total number of studies over the four timeframes, no significant differences were found (52.4% versus 61.9% versus 50.0% versus 47.5%), but the rate of phase II trials was 34.2% (68 of 199, ranging from 0 to 9 per year), with a significant uptrend over the 20-year period (4 trials in 2000–2005 versus 28 trials in 2015–2019, *p*=0.001).

Most (167, 83.9%) of the studies were multiinstitutional trials.

Overall, 62 of 199 (37.1%) evaluated one or more types of targeted/biological therapies. Interestingly, there was a significant increase of trials investigating targeted/biological therapies over the years, moving from only one trial in the 2000–2004 up to 36 trials in the 2015–2019 (1.6% versus 58.1%, *p*=0.0001). Few trials had specific molecular histology or biomarker selection criteria at enrollment time (*n* = 15, 7.5%), and most of them (*n* = 10, 66.7%) were performed between 2015 and 2019.

**Figure 4:**
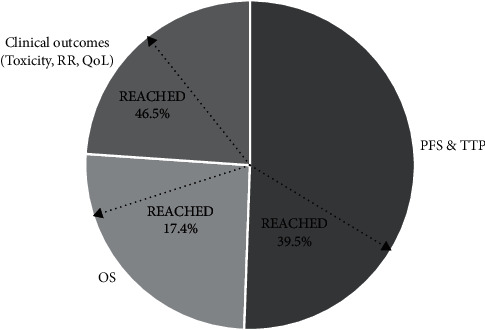
Primary outcomes of OC trials. ∗A single primary outcome was considered for each study. PFS, progression-free survival; TTP, time-to-progression; OS, overall survival; RR, response rate; QoL, quality of life.

Outcomes of interest were survival outcomes, including progression-free survival (PFS) and overall survival (OS), and clinical outcomes such as response rate, quality of life, and toxicity (Figure 4). To note, because of similar time-to-event definition, time-to-progression (TTP) was included in PFS evaluation.

In details, when PFS was the primary (*n* = 91, 45.7%) or the coprimary (*n* = 14, 7.0%) endpoint, it was reached in 37.1% of the cases (*n* = 39). Of note, histology or molecular biomarker selection criteria were associated with improved PFS outcome. In fact, among the 15 molecular/histology driven trials, 13 (86.7%) had PFS as the primary endpoint and 9 studies (69.2%) reported improved PFS results.

Advantage in OS was reported in 9 studies (4.5%, *n* = 8 phase III, *n* = 1 phase II). No study with histology/molecular assessment evaluated OS as primary endpoint.

Finally, over the last years, the use of quality of life as primary endpoint has increased (*n* = 3 out of 61 trials, 4.9%).

Based on clinical scenarios, (i) surgery; (ii) chemotherapy and maintenance therapy in the primary setting; and (iii) chemotherapy and maintenance therapy in the recurrent setting, 6 trials (50.0%), 29 trials (30.9%), and 37 trials (39.8%) met the primary endpoint, respectively. Benefit in PFS has been shown in trials testing chemotherapy and maintenance therapy in both primary (*n* = 18, 62.1%) and recurrent (*n* = 21, 56.8%) setting, whereas there was no positive PFS outcome in surgical studies. In the surgical setting, positive endpoints reached were OS (*n* = 1, 16.7%) and clinical outcomes (response rate = 3, 50%; toxicity = 2, 33.3%).

Considering clinical outcomes, there was a consistent improvement in positive studies over the 20-year period, from three trials in 2000–2004 up to 15 trials in 2015–2019. This improvement was strictly associated to a higher proportion of positive studies testing target therapy (31.3% in 2015–2019).

## 4. Discussion

This review represents a synthesis of the available data on published trials in OC and a basis for further considerations. In the last 20 years, median OS of OC patients moved from expected 3 years to more than 5 years [5, 6]. Several reasons can explain this relevant increase, including the improvement of surgical skills as well as the identification of referral centers for OC management. Moreover, the development of targeted therapeutic options (for instance PARP-inhibition) could be considered as a leading actor in the overall improvement in survival of ovarian cancer patients over the last two decades. Nonetheless, both the design of high-quality trials and the better assessment of OC biomolecular profile have certainly led to this encouraging progress.

Accordingly, in our analysis of clinical trials over the last two decades, we found a significant increase in the proportion of studies reporting positive outcomes, particularly PFS, despite a slight overall decrease of patients enrolled per study. In other words, albeit the number of performed studies had a 50% increase, less women have been enrolled in these studies, and more successful trials have been carried out, time by time. This suggests that studies have become more efficient at producing positive results, without involving unnecessary patients. We therefore tried to understand which have been the clues of this overall improvement.

First of all, we have to document that, in the OC research landscape, the importance of multicenter trials has been recognized since the early 2000s, as the number of multicenter trials ranges from 74% to 95% from the 2000–2004 to the 2015–2019 timeframes, with a slight reduction across the years.

As expected, new biological drugs have been increasingly developed and investigated in the last decade, with 21326 patients enrolled in studies investigating targeted therapies, compared with only 989 patients in 2000–2004 and 2005–2009 timeframes. Nonetheless, the introduction of targeted therapies alone did not completely explain the increase of reached endpoints, recorded over the years.

Otherwise, it can be reasonably hypothesized that this increase in successful trials has been allowed by the use of histology/molecular biomarkers in clinical trial design, which was peculiar of the last 5 years; in fact, from 2015–2019, more than 50% of studies reached the planned primary endpoint and 10 out of 61 trials were molecular-driven.

The greatest results have been found in the medical setting. Conversely, surgical studies were extremely few (only 6.0% of the included studies) and did not reach the planned endpoint in half of the cases. Moreover, no molecular-driven trials have been proposed in this background, even in the later timeframe. Our results therefore suggest that studies without preplanned patient selection might induce study's failure and a personalized approach should be proposed also for surgical trials.

Although few similar studies have been performed in other tumor settings such as the lung and sarcoma [7, 8], to the best of our knowledge, our analysis is the first to elucidate the overall landscape of OC trials over the last two decades [7, 8]. Interestingly, the advent of targeted/biological therapies and biomarker-driven trials has occurred later in OC, compared with other tumors [7, 8]. Nonetheless, when focusing on outcomes reached, we have obtained the most significant growth over the time, especially in term of PFS, probably due to the design of more personalized studies.

There are some limitations of this analysis. First, phase I studies were excluded because they are typically focused on the safety of systemic treatments and not on survival improvement, with PFS and OS outcomes. We also did not collect all data on study design because not relevant for our purposes; also, some studies, even with interesting results, have been presented at conferences but are still waiting for publication, and therefore, they have not been included in the present analysis. Furthermore, it should be considered that we stated as “positive studies” those in which primary outcome was reached, but it should be recognized that “reached outcome” does not always means “clinical-practice changing” trial. Moreover, this definition might be affected not only by local approval of drug's administration—Food and Drug Administration (FDA) or European Medicines Agency (EMA)—but also by guidelines endorsement. For instance, intraperitoneal chemotherapy and hyperthermic intraperitoneal chemotherapy are both valid options for advanced OC management according to National Comprehensive Cancer Network (NCCN) guidelines, but these strategies are not recommended by European Society of Gynaecological Oncology (ESGO) guidelines [2, 9]. Besides, it has to be noticed that “clinical-practice changing” trials could also be considered “negative studies” as they may answer many clinical questions not yet investigated. Actually, only approximately 50–60% of positive randomized control trials performed over the last 20 years have been significant for daily clinical practice; this further suggests that in several cases, methodology and studies results were well-designed, but rationale or research idea was probably too weak or inadequately tailored. Remarkably, among biomarker-driven studies, almost 90% (8/9) [10–18] were both positive and practice changing trials, underlying the importance of a personalized approach.

Despite these limitations, our data show provocative changes in the OC clinical trial landscape in the last 20 years and demonstrate that the incidence of clinical studies with positive survival and clinical outcomes is progressively increasing.

## 5. Conclusions

Our data confirm that efforts of the scientific community to improve OC patients management have been countless in the last 20 years, suggesting to persevere toward a more cost-effective, patient-oriented, and individualized fight against OC, in the medical but also in the surgical setting.

## Figures and Tables

**Table 1:** Studies characteristics.

Characteristics		Timeframe				*P*
		2000–2004	2005–2009	2010–2014	2015–2019	
Total studies	199	42	42	54	61	NA
Total patients	72934	16161	14054	23024	19695	0.45
Multicenter (%)	167 (83.9)	40 (95.2)	41 (97.6)	41 (75.9)	45 (73.8)	0.01
Phase III trials (%)	104 (52.3)	22 (52.4)	26 (61.9)	27 (50.0)	29 (47.5)	0.01
Topic						0.01
Surgery (%)	12 (6.0)	1 (2.4)	3 (7.1)	2 (3.7)	6 (9.8)	
Systemic therapy in the primary setting (%)	94 (47.2)	27 (64.3)	26 (61.9)	20 (37.0)	21 (34.4)	
Systemic therapy in the recurrent setting (%)	93 (46.7)	14 (33.3)	13 (31.0)	32 (59.3)	34 (55.7)	
Positive studies (%)	72 (36.2)	10 (23.8)	13 (31.0)	16 (29.6)	33 (54.1)	0.004
Histology or molecular biomarker selection criteria (%)	15 (7.5)	1 (2.4)	1 (2.4)	3 (5.6)	10 (16.4)	0.0001
Targeted therapy (%)	62 (31.2)	1 (2.4)	1 (2.4)	24 (44.4)	36 (59.0)	0.004

## Data Availability

The data used to support the findings of this study are available from the corresponding author upon request.
